# A clinical study of Simotang oral liquid combined with Shenque acupoint application for abdominal distension after cesarean section

**DOI:** 10.3389/fmed.2026.1920253

**Published:** 2026-09-11

**Authors:** Lijuan Du, Jianjun Hao, Fei Wang, Liyan Hu, Nannan Zhao, Caixia Yang

**Affiliations:** 1 Obstetrics Department, Shanxi Provincial Children’s Hospital, Shanxi Provincial Maternal and Child Health Care Hospital, Taiyuan, Shanxi, China; 2 Department of Traditional Chinese Medicine, Shanxi Provincial Children’s Hospital, Shanxi Provincial Maternal and Child Health Care Hospital, Taiyuan, Shanxi, China

**Keywords:** acupoint sticking therapy, cesarean section, gastrointestinal motility, parturients, postoperative ileus

## Abstract

**Objective:**

To evaluate the preventive and therapeutic effects of Simotang oral liquid combined with Shenque acupoint application on abdominal distension after cesarean section, and assess its safety.

**Methods:**

A prospective randomized controlled trial was conducted. Parturients who underwent elective cesarean section in our hospital from June 2024 to June 2025 were enrolled and randomly divided into the intervention group (treated with Simotang oral liquid combined with Shenque acupoint application) and the control group (receiving standard nursing care), with 100 cases in each group. Compare the postoperative recovery indicators of the two groups.

**Results:**

The median recovery time of bowel sounds [8 (6, 10) h vs. 12 (10, 14) h], median time to first flatus [20 (17, 26) h vs. 26 (24, 29.5) h], and median time to first defecation [50 (48, 52) h vs. 55 (52, 61) h] in the intervention group were all significantly shorter than those in the control group (*P* < 0.001). There was no significant between-group difference in the overall incidence of abdominal distension, whereas the severity of abdominal distension was significantly lower in the intervention group, with zero severe distension cases observed in the intervention group. The rate of colostrum secretion within 60 h after surgery was 93.00% in the intervention group, which was significantly higher than 74.00% in the control group (*P* < 0.05).

**Conclusion:**

Simotang oral liquid combined with Shenque acupoint application can effectively promote the recovery of gastrointestinal function, alleviate the severity of abdominal distension (though it cannot reduce the overall incidence of distension), and facilitate early lactation after cesarean section, and thus possesses good clinical application value.

## 1 Introduction

Abdominal distension is one of the most common complications following cesarean section in obstetric practice (1). Cesarean delivery causes direct and indirect irritation to the abdominal cavity, pelvic cavity and intestinal tract. Combined with the inhibitory effect of anesthetics, postoperative pain-induced sympathetic excitation and decreased physical activity, these factors commonly suppress gastrointestinal function in parturients in the early postoperative stage (2, 3). Such gastrointestinal motility disorders are mainly manifested as weakened or absent bowel sounds, delayed flatus and defecation, abdominal distension, nausea and even intestinal obstruction. These symptoms not only aggravate maternal discomfort and reduce postoperative well-being, but also delay wound healing, interfere with nutrient intake and impair early lactation, thereby prolonging hospital stay and increasing medical resource consumption (4–6). Given the persistently high cesarean section rates worldwide in recent years, effectively accelerating the recovery of gastrointestinal function, and reducing the incidence of abdominal distension and related complications have become key issues in perioperative obstetric management (7, 8).

Current interventions for the recovery of gastrointestinal function after cesarean section mainly include early postoperative ambulation, gum chewing, administration of prokinetic agents, and warm water foot bath (9, 10). Nevertheless, these approaches have certain limitations in clinical practice. For instance, some parturients cannot achieve effective early ambulation due to postoperative pain, physical fatigue or concomitant underlying diseases. Although prokinetic agents exert certain therapeutic effects, they may trigger adverse reactions such as nausea, diarrhea and cardiovascular side effects, and the safety of some of these drugs during lactation remains controversial (11, 12). Accordingly, exploring more effective, mild and well-tolerated interventions while ensuring safety is of great clinical significance.

Traditional Chinese medicine has accumulated abundant theoretical and practical experience in the regulation of postoperative gastrointestinal dysfunction. Postoperative abdominal distension after cesarean section is mostly attributed to stagnation of qi movement and obstruction of fu-qi. Surgical trauma consumes qi and blood, leading to dysfunction of zang-fu organs, among which impaired transportation and transformation of the spleen and stomach as well as disordered conduction of the large intestine are the main pathogenesis. The basic therapeutic principles include promoting qi movement to resolve stagnation, unblocking fu-organs and descending adverse qi, and regulating the spleen and stomach. Derived from Simo Yin recorded in Jisheng Fang of the Song Dynasty, Simotang oral liquid is composed of Aucklandiae Radix, Aurantii Fructus Immaturus, Arecae Semen and Linderae Radix. It exerts the effects of promoting qi movement and descending adverse qi, soothing the middle energizer and relieving distension, and is widely used clinically for qi stagnation syndromes such as postoperative abdominal distension, functional dyspepsia and constipation. Modern pharmacological studies have demonstrated that Simotang can promote the contraction of gastrointestinal smooth muscle, enhance intestinal peristalsis and improve gastrointestinal blood flow, thereby accelerating the recovery of postoperative gastrointestinal function (13, 14). Located at the umbilicus, Shenque acupoint is an important acupoint of the Conception Vessel, connecting with the meridians of the whole body and internally linking zang-fu organs, especially the spleen, stomach, small intestine and large intestine. Acupoint application refers to applying ground traditional Chinese medicinal materials to Shenque acupoint. It exerts the effects of regulating qi movement, warming and unblocking meridians, harmonizing the stomach and unblocking fu-organs through the dual actions of drug permeation and acupoint stimulation. This therapy features simple operation, non-invasiveness and painlessness, and is highly acceptable among parturients. In addition, transdermal drug absorption can avoid the first-pass effect of the liver and reduce the risk of systemic adverse reactions (15).

Based on the above theories, Simotang oral liquid and Shenque acupoint application exert synergistic effects via different mechanisms: oral medication regulates gastrointestinal function systematically, while external therapy acts directly on the gastrointestinal system through local acupoint stimulation. The combined use of the two interventions may achieve the comprehensive advantages of integrated oral and external treatment that addresses both the root cause and clinical symptoms. Therefore, conducting a clinical study on the preventive and therapeutic effects of Simotang oral liquid combined with Shenque acupoint application on abdominal distension after cesarean section can not only enrich the theoretical basis for integrated traditional Chinese and Western medicine in perioperative management, but also provide a more optimized intervention strategy for clinical practice.

## 2 Materials and methods

### 2.1 Study subjects

This study adopted a prospective randomized controlled trial design. Parturients who underwent elective cesarean section at our hospital between June 2024 and June 2025 were enrolled as research subjects. They were divided into the intervention group receiving Simotang oral liquid combined with Shenque acupoint application and the control group receiving standard nursing care by the random number table method, with 100 participants in each group and a total of 200 cases. All enrolled parturients underwent elective cesarean section under combined spinal-epidural anesthesia; general anesthesia was not performed in any case. The standardized spinal-epidural anesthesia regimen was uniformly applied to all participants in both groups to avoid the confounding influence of different anesthesia types on intestinal motility recovery.

The study protocol was reviewed and approved by the Ethics Committee of our hospital (Approval No.: IRB-KYYN-2024-004), and written informed consent was obtained from all participants or their family members.

Inclusion criteria: ① Aged 20 to 40 years old; ② Full-term pregnancy with gestational age ≥ 37 weeks; ③ Singleton pregnancy and cephalic presentation; ④ Elective lower segment cesarean section; ⑤ No preoperative gastrointestinal motility disorders and no history of abdominal surgery; ⑥ No severe cardiac, hepatic, renal or other organic diseases, as well as no pregnancy complications; ⑦ No history of allergy to the medications used in this study; ⑧ Voluntary participation in this study with signed informed consent.

Exclusion criteria: ① Parturients undergoing emergency cesarean section or experiencing severe intraoperative complications; ② Parturients with preoperative gastrointestinal abnormalities such as intestinal obstruction, abdominal distension and diarrhea; ③ Parturients suffering from severe complications including massive hemorrhage and infection during or after surgery; ④ Parturients with allergies to ingredients of Simotang oral liquid or topical medicines for acupoint application; ⑤ Cases with local skin damage, infection or dermatoses at the Shenque acupoint; ⑥ Participants with mental illness or cognitive impairment who were unable to cooperate with the study; ⑦ Parturients whose newborns required transfer to the pediatric department for treatment.

Based on the results of the preliminary trial, it was estimated that the time to first postoperative flatus in the intervention group would be reduced by approximately 20% compared with the control group. With a two-sided *α* of 0.05 and a statistical power (1-*β*) of 0.80, and accounting for a 20% loss to follow-up, the minimum required sample size was calculated as 45 cases per group. A total of 100 participants were finally enrolled in each group, with 200 cases in total.

### 2.2 Grouping methods

Participants were allocated using the random number table method. Research staff who did not participate in subsequent interventions and outcome assessments generated random sequences via statistical software, and the corresponding serial numbers were placed in opaque sealed envelopes. When a subject met the inclusion criteria, the envelope with the matching number was opened in the order of enrollment, and the participant was assigned to either the intervention group or the control group according to the allocation result. This procedure ensured adequate allocation concealment.

Data collectors were not informed of group assignment before outcome assessment. However, given the obvious difference between the combined oral and external intervention and standard nursing care, participants, ward nurses and data collectors could readily distinguish group allocation during follow-up, making this an open-label trial with no blinding of participants, caregivers or outcome assessors.

Both groups received unified partial ERAS perioperative nursing protocols including standardized anesthesia, intravenous fluid management, analgesia and early mobilization. However, the full ERAS fast-track 24-hour discharge pathway was not adopted in this study; all participants followed the conventional domestic obstetric discharge criteria in China instead.

#### 2.2.1 Control group

Participants in the control group received standard nursing care, which included unified partial ERAS perioperative protocols and routine nursing care interventions. The specific contents of routine nursing care interventions were as follows: pillow-free supine rest for 6 h after surgery; assisted turning at 6 h and encouragement of early ambulation; small amounts of liquid food (rice soup, warm water) available 6 h postoperatively, followed by a gradual shift to semi-liquid and regular diet after first flatus. Intravenous fluid infusion, anti-infection and analgesic treatments were administered per medical orders. To avoid mutual interference between two interventions, participants of the control and intervention groups were housed in separate wards.

#### 2.2.2 Intervention group

On the basis of the standard nursing care provided to the control group, participants in the intervention group additionally received combined treatment with Simotang oral liquid and Shenque acupoint application. (1) Simotang oral liquid: The commercially available Simotang oral liquid was used, with main ingredients including Aucklandiae Radix, Aurantii Fructus Immaturus, Arecae Semen and Linderae Radix. Oral administration was initiated 6 h after surgery at a dose of 20 mL twice daily, and the medication was continued until the first defecation or for a maximum of 5 consecutive days. (2) Shenque acupoint application: Traditional Chinese medicinal materials including Caryophylli Flos, Evodiae Fructus and Cinnamomi Cortex were ground into powder and mixed with ginger juice or honey to form a paste. The paste was made into medicinal plasters with a diameter of approximately 2 cm and placed in the center of sterile dressings. The plasters were applied to Shenque acupoint (umbilicus) 6 h after surgery for 6 to 8 h each time, once a day, for 3 consecutive days. During the application period, local skin conditions were closely monitored for adverse reactions such as redness, pruritus and allergy, and timely interventions were performed once any discomfort occurred.

### 2.3 Observation indicators and evaluation criteria

Within 3 days of patient enrollment, the responsible nurse collects the patient's general information by reviewing medical records (Age, gestational age, body mass index, surgery time, pregnancy history). Two researchers who have undergone unified training independently collect clinical data and cross-check to ensure data accuracy.

#### 2.3.1 Primary observation indicators

1. Recovery time of bowel sounds: The attending nurses auscultated abdominal bowel sounds every 2 h. Bowel sound recovery was defined as more than 3 bowel sounds per minute, and the time (in hours) from the end of surgery to bowel sound recovery was recorded.
2. Time to first flatus: The time (in hours) from the end of surgery to the first spontaneous flatus of parturients was recorded.
3. Time to first defecation: The time (in hours) from the end of surgery to the first defecation of parturients was recorded.

#### 2.3.2 Secondary observation indicators

1. Incidence and severity of abdominal distension: Evaluation was performed according to the grading criteria for abdominal distension. Grade 0 (no abdominal distension): abdominal circumference increased by less than 2 cm without subjective distension; Grade I (mild abdominal distension): slight abdominal wall tension and abdominal protuberance with no impact on sleep; Grade II (moderate abdominal distension): increased abdominal wall tension and moderate abdominal protuberance accompanied by nausea, which greatly affected sleep; Grade III (severe abdominal distension): marked abdominal wall tension and obvious abdominal protuberance with tympanic sound on auscultation, together with vomiting, restlessness and groaning that disturbed sleep. The incidence of abdominal distension and the distribution of different severity grades in each group were statistically analyzed.
2. Colostrum secretion: Colostrum secretion of parturients was assessed at 48, 60 and 72 h after surgery, and the time to initial lactation was recorded.
3. Postoperative length of hospital stay: The number of days from surgery to hospital discharge was documented.
4. Incidence of postoperative intestinal obstruction: The occurrence of postoperative intestinal obstruction was counted in both groups. The diagnostic criteria for intestinal obstruction included obvious abdominal distension, cessation of flatus and defecation, and presence of air-fluid levels on abdominal plain radiographs.
5. Adverse reactions and events: The occurrence of adverse reactions such as nausea, diarrhea, skin rash and allergy during the intervention was observed and recorded in the two groups.

Hospital discharge complied with standardized local obstetric criteria, including stable maternal vital signs, intact incision recovery, successful oral feeding, established lactation and normal neonatal jaundice screening results, rather than 24-hour fast-track ERAS discharge standards.

### 2.4 Statistical analysis

SPSS 26.0 software was used for data analysis. The normality of the data is assessed using a Q-Q plot. Normally distributed measurement data were presented as mean ± standard deviation (x ± s), and between-group comparisons were performed using the independent-samples t test. Non-normally distributed measurement data were expressed as median (interquartile range[IQR]), and the Mann–Whitney U test was adopted for intergroup comparison. Enumeration data were described as frequency (percentage[%]), and the chi-square test or Fisher's exact test was used for group comparisons. All statistical tests were two-tailed, and a *P* value less than 0.05 was considered statistically significant.

## 3 Results

### 3.1 Comparison of clinical data

A total of 200 parturients undergoing elective cesarean section were enrolled in this study, including 100 cases in the intervention group receiving Simotang oral liquid combined with Shenque acupoint application and 100 cases in the control group receiving standard nursing care. Comparison of baseline characteristics revealed that the two groups were well balanced in terms of age, gestational age, body mass index, operation duration and obstetric history. The median age was 30 (28, 33) years in the intervention group and 30 (28, 32) years in the control group, with no statistically significant difference (*P* > 0.05). The median gestational age was 39.10 (38.40, 39.40) weeks in the intervention group and 39.00 (38.40, 39.40) weeks in the control group, showing no significant between-group difference (*P* > 0.05). The body mass index was 21.86 (20.31, 23.84) kg/m^2^ in the intervention group and 21.70 (20.45, 23.44) kg/m^2^ in the control group, without statistical difference (*P* > 0.05). The operation duration was 35.12 ± 0.49 min in the intervention group and 34.95 ± 0.59 min in the control group, and no significant difference was observed between the two groups (*P* > 0.05). With regard to obstetric history, there were 30 multiparous parturients (30.00%) in the intervention group and 38 multiparous parturients (38.00%) in the control group, and the constituent ratios presented no statistically significant difference (*P* > 0.05). The above results indicated that the baseline data were comparable between the two groups. Detailed data are shown in Table 1.

**Table 1:** Comparison of baseline characteristics of parturients between the two groups.

Item	Intervention group (*n* = 100)	Control group (*n* = 100)	Z/t/*χ*^2^	*P* value
Age (years)	30 (28, 33)	30 (28, 32)	−0.385	0.701
Gestational age (weeks)	39.10 (38.40, 39.40)	39.00 (38.40, 39.40)	0.520	0.603
Body mass index (kg/m^2^)	21.86 (20.31, 23.84)	21.70 (20.45, 23.44)	−0.182	0.856
Operation duration (min)	35.12 ± 0.49	34.95 ± 0.59	−0.221	0.825
Primipara/multipara (cases)	70/30	62/38	1.426	0.232

Non-normal continuous variables (age, gestational age, BMI) were expressed as median (interquartile range) and analyzed by Mann–Whitney U test, with Z values presented. Normally distributed continuous variable (operation duration) was presented as mean ± standard deviation and compared using independent-samples t test. Enumeration data of parity were compared by chi-square test.

### 3.2 Comparison of gastrointestinal function recovery indicators between the two groups

In terms of gastrointestinal function recovery, the recovery time of bowel sounds, time to first flatus and time to first defecation after surgery were all significantly shorter in the intervention group than in the control group. Specifically, the median recovery time of bowel sounds was 8 (6, 10) hours in the intervention group and 12 (10, 14) hours in the control group, representing an approximately 4-hour reduction in the intervention group with a statistically significant difference (*P* < 0.001). The median time to first flatus was 20 (17, 26) hours in the intervention group and 26 (24, 29.5) hours in the control group, with flatus occurring about 6 h earlier in the intervention group (*P* < 0.001). The median time to first defecation was 50 (48, 52) hours in the intervention group and 55 (52, 61) hours in the control group, indicating defecation occurred roughly 5 h earlier in the intervention group (*P* < 0.001). These findings suggest that Simotang oral liquid combined with Shenque acupoint application can markedly accelerate the early recovery of gastrointestinal function after cesarean section. Detailed data are presented in Table 2.

**Table 2:** Comparison of postoperative clinical indicators and safety profiles between the two groups.

Indicator	Intervention group (*n* = 100)	Control group (*n* = 100)	t/χ^2^	*P* value
Gastrointestinal function recovery				
Time to recovery of bowel sounds (h)	8 (6, 10)	12 (10, 14)	8.762	< 0.001
Time to first flatus (h)	20 (17, 26)	26 (24, 29.5)	6.253	< 0.001
Time to first defecation (h)	50 (48, 52)	55 (52, 61)	7.168	< 0.001
Abdominal distension				
Overall incidence of abdominal distension [n (%)]	17 (17.00)	12 (12.00)	1.008	0.315
Grade 0 (No distension) [n (%)]	83 (83.00)	88 (88.00)		
Grade I (Mild distension) [n (%)]	16 (16.00)	1 (1.00)		
Grade II (Moderate distension) [n (%)]	1 (1.00)	2 (2.00)		
Grade III (Severe distension) [n (%)]	0 (0.00)	9 (9.00)		
Colostrum secretion			18.110	< 0.001
Colostrum secretion within 48 h postoperatively [n (%)]	9 (9.00)	1 (1.00)		
Colostrum secretion within 60 h postoperatively [n (%)]	84 (84.00)	73 (73.00)		
Colostrum secretion within 72 h postoperatively [n (%)]	7 (7.00)	26 (26.00)		
Prognosis and safety				
Postoperative hospital stay (d)	4 (4, 5)	4 (4, 5)	−0.100	0.920
Postoperative intestinal obstruction [n (%)]	0 (0.00)	1 (1.00)	-	-
Overall incidence of adverse reactions [n (%)]	0 (0.00)	1(1.00)	-	-

Time and hospital stay data were non-normal, expressed as median (IQR) and compared via Mann–Whitney U test (Z values). Chi-square test was used for overall distension incidence and colostrum distribution; Fisher's exact test for rare binary adverse events. Graded distension and stratified lactation data were presented descriptively without separate statistics. Adverse reactions included nausea, diarrhea and umbilical skin irritation from acupoint application, all mild and manageable.

### 3.3 Comparison of abdominal distension between the two groups

Regarding the incidence of postoperative abdominal distension, the overall rate was 17.00% (17/100) in the intervention group and 12.00% (12/100) in the control group, with no statistically significant difference between the two groups (*P* > 0.05). In terms of the distribution of distension severity grades, there were 83 cases with no distension (Grade 0, 83.00%), 16 cases with mild distension (Grade Ⅰ, 16.00%), 1 case with moderate distension (Grade Ⅱ, 1.00%) and 0 cases with severe distension (Grade Ⅲ, 0.00%) in the intervention group. In the control group, 88 cases had no distension (Grade 0, 88.00%), 1 case had mild distension (Grade Ⅰ, 1.00%), 2 cases had moderate distension (Grade Ⅱ, 2.00%) and 9 cases (Grade Ⅲ, 9.00%) had severe distension. Although the overall incidence of abdominal distension was not significantly different between the two groups, severe abdominal distension was only observed in the control group. This indicates that the combined intervention can effectively mitigate the severity of abdominal distension rather than reducing its overall incidence. Detailed data are shown in Table 2.

### 3.4 Comparison of colostrum secretion between the two groups

With respect to colostrum secretion, 9 participants (9.00%) in the intervention group produced colostrum within 48 h after surgery, 84 participants (84.00%) within 60 h, and 7 participants (7.00%) within 72 h. In the control group, 1 participant (1.00%) produced colostrum within 48 h, 73 participants (73.00%) within 60 h, and 26 participants (26.00%) within 72 h. There was a statistically significant difference in the distribution of colostrum secretion time between the two groups (*P* < 0.01). The proportion of participants who achieved lactation within 60 h postoperatively was markedly higher in the intervention group, suggesting that the combined intervention facilitates early lactation. This effect may be associated with improved gastrointestinal function and the early recovery of nutrient intake. Detailed data are presented in Table 2.

### 3.5 Comparison of postoperative hospital stay, intestinal obstruction and adverse reactions between the two groups

The median postoperative hospital stay was 4 (4, 5) days in both the intervention group and the control group, with no statistically significant difference (*P* > 0.05). No case of postoperative intestinal obstruction was observed in the intervention group, while one case occurred in the control group. In terms of safety assessment, no systemic adverse reactions or local cutaneous reactions (including umbilical erythema, pruritus, rash and contact allergy related to acupoint application) were observed in the intervention group. No skin adverse events were noted in the control group either, and only one participant in the control group developed mild nausea. Detailed data are presented in Table 2.

## 4 Discussion

The results of this study demonstrated that combined application of Simotang oral liquid and Shenque acupoint application can significantly promote the recovery of gastrointestinal function after cesarean section. This combined therapy significantly shortens bowel sound recovery time, time to first flatus and time to first defecation, alleviates the severity of abdominal distension and promotes early colostrum secretion with good safety. No significant intergroup differences were found in the overall incidence of abdominal distension or postoperative hospital stay.

The mechanisms underlying gastrointestinal dysfunction after cesarean section are complex, involving multiple factors such as the inhibitory effect of anesthetics on intestinal nerves, surgical trauma-induced stress response, increased sympathetic nerve excitability and release of inflammatory mediators. Derived from Simo Yin recorded in Jisheng Fang of the Song Dynasty, Simotang oral liquid is composed of Aucklandiae Radix, Aurantii Fructus Immaturus, Arecae Semen and Linderae Radix, and exerts the effects of promoting qi movement and descending adverse qi, soothing the middle energizer and relieving distension. Modern pharmacological studies have revealed that Simotang can stimulate the contraction of gastrointestinal smooth muscle, enhance intestinal peristalsis and improve gastrointestinal blood flow (16). Pang YT reported that Simotang combined with acupoint massage after cesarean section significantly shortened the time to bowel sound recovery, relief of abdominal distension, first flatus, and first defecation compared with routine treatment (17). These findings are highly consistent with our results, further confirming the key role of Simotang oral liquid in facilitating the recovery of gastrointestinal function after cesarean section. Zhou YC et al. demonstrated in a randomized, double-blind, placebo-controlled trial that Simotang significantly shortened the time to bowel sound recovery, first flatus, and first defecation after gynecological laparotomy, providing further evidence for its postoperative gastrointestinal pro-motility effect (18). Collectively, these studies indicate that Simotang can effectively restore gastrointestinal function in parturients after cesarean section by promoting qi circulation, eliminating stagnation and accelerating intestinal peristalsis.

As an important external therapy of traditional Chinese medicine, Shenque acupoint application deserves further exploration regarding its mechanism of action. Located at the umbilicus, Shenque acupoint is an essential acupoint of the Conception Vessel. It connects to the meridians throughout the body and links internally to zang-fu organs, especially the spleen, stomach, small intestine and large intestine. Through the dual effects of drug permeation and acupoint stimulation, acupoint application can regulate qi movement, warm and unblock meridians, harmonize the stomach and unblock fu-organs. A study by Lu SJ et al. demonstrated that traditional Chinese medicine acupoint application at Shenque and Zusanli combined with TDP lamp irradiation could effectively improve gastrointestinal function recovery after cesarean section. The treatment group presented earlier recovery of bowel sounds, flatus and defecation than the control group (*P* < 0.05). The incidence of abdominal pain and distension on postoperative day 4 was 5.00% in the treatment group, which was significantly lower than 16.25% in the control group (*P* < 0.05) (19). Another study by Li JL et al. further confirmed that the combined use of Simotang and acupoint application with qi-regulating prescriptions in post-cesarean parturients led to markedly shorter time to bowel sound recovery, first flatus and first defecation in the observation group. The incidence of postoperative abdominal distension was only 3.3% in the observation group, obviously lower than 20.0% in the control group (*P* < 0.05) (20). All the above studies support the beneficial effect of acupoint application on accelerating postoperative gastrointestinal function recovery.

The combined application of Simotang oral liquid and Shenque acupoint application in this study yielded superior outcomes compared with monotherapy. This finding can be explained by the traditional Chinese medicine principle of integrated oral and external treatment that addresses both the root cause and clinical symptoms. Orally administered Simotang regulates qi movement and relieves stagnation systematically to exert whole-body effects. Shenque acupoint application acts directly on the gastrointestinal system via local acupoint stimulation and produces topical effects. Their synergistic action not only enhances intestinal peristalsis but also alleviates local distension symptoms, thereby achieving better clinical efficacy.

From an integrated Chinese and Western medicine perspective, postoperative abdominal distension is rooted in TCM qi stagnation and fu-qi obstruction, and the qi-regulating, fu-unblocking efficacy of our combined intervention can be interpreted through three measurable physiological pathways supported by clinical evidence (21). First, qi stagnation corresponds to disordered secretion of gastrointestinal motility hormones. Qi-regulating herbal formulas such as Simotang significantly elevate serum motilin and gastrin while reducing vasoactive intestinal peptide (VIP), which strengthens intestinal smooth muscle contraction and accelerates bowel sound recovery, first flatus and defecation, consistent with our objective intestinal recovery outcomes (21). Second, relieved abdominal distension and somatic discomfort reduce postoperative sympathetic overactivation, which removes the inhibitory effect on endogenous oxytocin and prolactin secretion; elevated oxytocin promotes mammary gland contraction and early colostrum secretion, explaining the higher 60-hour lactation rate observed in the intervention group (4, 6). Third, severe abdominal distension aggravates surgical pain and increases postoperative opioid analgesic consumption, whereas opioids further suppress intestinal peristalsis and worsen qi stagnation to form a vicious cycle (15, 19). By mitigating distension and pain, this combined TCM regimen may lower cumulative opioid requirements and break this inhibitory cycle. It should be clarified that we did not collect serum motilin, oxytocin levels or total postoperative opioid consumption in this trial, so the above mechanistic correlations are inferred from existing published data; further trials incorporating these biomarkers are required to provide direct laboratory evidence validating the qi stagnation pathological mechanism.

From the perspective of modern medical mechanisms, a study by Fang H demonstrated that a self-formulated Paiqi Decoction significantly increased serum gastrin (GAS) and motilin (MTL) levels while decreasing VIP, thereby facilitating gastrointestinal function recovery after cesarean section (21). These findings provide a reference for understanding the mechanisms by which combined Simotang oral liquid and acupoint application improve gastrointestinal function in the present study. Meanwhile, studies on external application of Evodiae Fructus powder at the Shenque acupoint combined with acupoint massage have also confirmed that topical application of traditional Chinese medicines at Shenque can effectively promote flatus and defecation, and accelerate the early recovery of gastrointestinal function after cesarean section.

This study also found that the proportion of participants with colostrum secretion within 60 h after surgery was significantly higher in the intervention group than in the control group, which is consistent with the conclusion reported by Chen CL et al. Their study also showed that the rate of early colostrum secretion was higher in the treatment group (*P* < 0.05) (17). Earlier colostrum secretion observed in the intervention group is linked to restored gastrointestinal function via multi-layered physiological, TCM theoretical and ERAS-aligned mechanisms rather than merely accelerated nutrient intake. From a modern obstetric perspective, relieved postoperative abdominal distension reduces sympathetic hyperarousal and opioid analgesic demand, removing central inhibition of oxytocin and prolactin secretion to facilitate the milk ejection reflex; meanwhile, early recovery of bowel motility enables timely enteral feeding to supply nutritional substrates for mammary milk synthesis, forming a bidirectional gut-lactation regulator*y* axis supported by recent endocrine research (22). TCM theory further complements this association: milk originates from food qi and blood transformed by the spleen-stomach, and the Foot-Yangming Stomach Meridian directly distributes to the breast; postoperative qi stagnation and fu-organ obstruction disrupt spleen-stomach transportation, while combined oral-external TCM therapy unblocks stagnation and replenishes milk's biochemical source, consistent with pooled evidence from TCM adjunct lactation trials post-cesarean section (23). Importantly, this integrated Simotang and Shenque acupoint regimen closely aligns with core obstetric ERAS goals, which prioritize early gut recovery, minimal opioids and prompt breastfeeding initiation; ERAS randomized trials confirm that interventions accelerating intestinal rehabilitation consistently raise early lactation rates, making this dual TCM therapy a valuable supplementary strategy to standard fast-track perioperative protocols for cesarean delivery (24). Additional animal research further validates that intestinal adaptive changes driven by restored gastrointestinal motility directly underpin maternal lactational nutrient allocation capacity after surgery (25).

From the perspective of pathophysiological mechanisms, early recovery of postoperative gastrointestinal function enables parturients to resume oral intake sooner, improve nutritional status, and promote the secretion and release of prolactin. Meanwhile, relief from discomforts such as abdominal distension and pain facilitates early maternal-neonatal contact and promotes the establishment of the lactation reflex. In the study by Chen JJ et al., the traditional Chinese medicine syndrome scores including epigastric pain and abdominal distending pain were significantly lower in the group treated with self-formulated qi-regulating decoction than in the control group (*P* < 0.05) (21). This indicates that herbal intervention not only improves objective gastrointestinal parameters but also alleviates subjective symptoms, which provides indirect evidence for the improvement of lactation observed in our study.

In terms of safety evaluation, there were no statistically significant difference between the two groups (*P* > 0.05). In the study conducted by Li JL et al., no severe complications such as intestinal obstruction occurred in either group after surgery (20). In another study by Fang H et al., the overall incidence of adverse reactions was 6.67% in the observation group, which was slightly lower than 9.33% in the control group, and the difference was not statistically significant (*P* > 0.05) (21). Collectively, these studies indicate that the combined use of Simotang oral liquid and Shenque acupoint application has a favorable safety profile. In the present trial, we closely monitored umbilical skin status throughout acupoint application for erythema, itching, rash and other topical allergic reactions; no local skin irritation or hypersensitivity events were recorded in the intervention group during the 3-day external treatment, further verifying the cutaneous safety of this combined regimen.

This study has several limitations. First, it was a single-center trial. Multi-center studies are needed in the future to verify the reliability of our findings. Second, objective indicators including GAS, MTL and VIP were not measured. The discussion on the underlying mechanisms was mainly based on published literature, without direct laboratory evidence. A previous study by Fang H et al. has confirmed that herbal interventions can significantly regulate the levels of these hormones (21). Further research should include the detection of such biomarkers to explore the mechanisms of the combined therapy in depth. In addition, only short-term clinical outcomes were observed in this study, with a lack of long-term follow-up data. Extended follow-up focusing on long-term recovery of parturients is recommended in subsequent investigations. Third, this single-center trial was conducted in a Chinese hospital with integrated TCM-WM obstetric service. Acupoint application and Simotang oral liquid may not be accessible in countries lacking TCM practitioners and herbal medicines, limiting its worldwide direct application. Multi-center trials covering different ethnic populations and medical systems are required to verify external validity. Fourth, postoperative length of stay followed local routine discharge criteria in China rather than international fast-track ERAS standards, which explains the longer hospitalization compared with Western 1-day discharge protocols. Fifth, this open-label trial only adopted standard nursing care as control, without placebo oral liquid or sham acupoint application. Unblinded grouping may bring expectation bias to subjective outcomes like abdominal distension severity and colostrum secretion; further blinded sham-controlled trials are required.

## 5 Conclusion

In conclusion, this study demonstrates that combined treatment of Simotang oral liquid and Shenque acupoint application effectively accelerates gastrointestinal function recovery, mitigates the severity of postoperative abdominal distension and promotes early colostrum secretion after cesarean section. This integrated oral-external TCM regimen possesses clinical application value in integrative obstetric settings.

## Data Availability

The original contributions presented in the study are included in the article/Supplementary Material, further inquiries can be directed to the corresponding author.

## References

[1] Agah J Baghani R Rakhshani MH Rad A. Metoclopramide role in preventing ileus after cesarean, a clinical trial. Eur J Clin Pharmacol. (2015) 71(6):657–62. 10.1007/s00228-015-1845-8 25877021

[2] Dimopoulos A Trompoukis A Christakopoulos D Kavrochorianos S Tsiampa E. Late-Onset paralytic ileus following cesarean section: a report of a rare case. Cureus. (2025) 17(4):e81954. 10.7759/cureus.81954 40351946 PMC12063509

[3] Charoenkwan K Nantasupha C Muangmool T Matovinovic E. Early versus delayed oral feeding after major gynaecologic surgery. Cochrane Database Syst Rev. (2024) 8(8):CD004508. 10.1002/14651858.CD004508.pub5 39132743 PMC11318081

[4] Salman L Aharony S Shmueli A Wiznitzer A Chen R Gabbay-Benziv R. Urinary bladder injury during cesarean delivery: maternal outcome from a contemporary large case series. Eur J Obstet Gynecol Reprod Biol. (2017) 213:26–30. 10.1016/j.ejogrb.2017.04.007 28411456

[5] Safrai M Stern S Gofrit ON Hidas G Kabiri D. Urinary tract injuries during cesarean delivery: long-term outcome and management. J Matern Fetal Neonatal Med. (2022) 35(18):3547–54. 10.1080/14767058.2020.1828336 33016166

[6] Chill HH Karavani G Reuveni-Salzman A Lipschuetz M Shimonovitz T Cohen N. Urinary bladder injury during cesarean delivery: risk factors and the role of retrograde bladder filling. Int Urogynecol J. (2021) 32(7):1801–6. 10.1007/s00192-020-04630-9 33386865

[7] Jensen AS Heinemeier IIK Schroll JB Rudnicki M. Iatrogenic bladder injury following gynecologic and obstetric surgery: a systematic review and meta-analysis. Acta Obstet Gynecol Scand. (2023) 102(12):1608–17. 10.1111/aogs.14641 37552010 PMC10619603

[8] Rao D Yu H Zhu H Duan P. The diagnosis and treatment of iatrogenic ureteral and bladder injury caused by traditional gynaecology and obstetrics operation. Arch Gynecol Obstet. (2012) 285(3):763–5. 10.1007/s00404-011-2075-7 21909750

[9] Wen Z Shen M Wu C Ding J Mei B. Chewing gum for intestinal function recovery after caesarean section: a systematic review and meta-analysis. BMC Pregnancy Childbirth. (2017) 17(1):105. 10.1186/s12884-017-1286-8 28415967 PMC5394625

[10] Gao T Liu Q Wang H Liu J Jin Y Liu J. The effects of coffee versus gum chewing after cesarean on bowel functions: a systematic review and network meta-analysis. Int J Surg. (2025) 111(11):8435–46. 10.1097/JS9.0000000000002911 40607963 PMC12626617

[11] Sinz S Warschkow R Tarantino I Steffen T. Gum chewing and coffee consumption but not caffeine intake improve bowel function after gastrointestinal surgery: a systematic review and network meta-analysis. J Gastrointest Surg. (2023) 27(8):1730–45. 10.1007/s11605-023-05702-z 37277676 PMC10412511

[12] Yenigul NN Aydogan Mathyk B Aslan Cetin B Yazici Yilmaz F Ayhan I. Efficacy of chewing gum for improving bowel function after cesarean sections: a randomized controlled trial. J Matern Fetal Neonatal Med. (2020) 33(11):1840–5. 10.1080/14767058.2018.1531122 30606082

[13] Wang XM Zhu WT Xu LC. Establishment of GC characteristic chromatogram for volatile components of simotang oral liquid and determination of the content of four components. Zhongguo Zhong Yao Za Zhi. (2023) 48(2):555–61. 10.19540/j.cnki.cjcmm.20220905.501 36725245

[14] Hu Y Bai Y Hua Z Yang J Yang H Chen W. Effect of Chinese patent medicine Si-Mo-Tang oral liquid for functional dyspepsia: a systematic review and meta-analysis of randomized controlled trials. PLoS One. (2017) 12(2):e0171878. 10.1371/journal.pone.0171878 28199409 PMC5310891

[15] Chae SY Sung WS Kim EJ Park SS. The effectiveness and safety of cupping therapy on CV8 shenque for Urticaria: a systematic review and meta-analysis. Altern Ther Health Med. (2025) 31(2):49–55. 10.2147/JPR.S435991 39715564

[16] Hu W Zhang H. Si Mo Yin Zi in treatment of type 2 diabetic nephropathy with elderly patients. Acta Chin Med. (2018) 33(10):1912–6.

[17] Pang YT. Effect of Chinese herbal decoction combined with acupoint massage on gastrointestinal function after cesarean section. Clin J Chin Med. (2019) 11(2):97–9.

[18] Zhou YC Huo JR Liu B Du J. The effect of Simotang oral solution on the gynecological laparotomy postoperative gastrointestinal motor function. J Clin Pathol Res. (2016) 36(11):1705–11. 10.3978/j.issn.2095-6959.2016.11.004

[19] Huang XM Lu SJ. Effect of traditional Chinese medicine acupoint application combined with TDP lamp irradiation on anal exhaust after cesarean section. Chin. Nurs. Res. (2017) 31(14):1779–80.

[20] Li JL Cai GQ Huang HL. Clinical application of Simotang combined with Paiqi prescription acupoint application for gastrointestinal function recovery after cesarean section. Clin J Chin Med. (2018) 10(17):29–31.

[21] Fang H. Effects of Paiqi Decoction on gastrointestinal dysfunction recovery and gastrointestinal hormone levels after cesarean section. Chin J Tradit Med Sci Technol. (2023) 30(1):74–7.

[22] Taylor VJ. Lactation from the inside out: maternal homeorhetic gastrointestinal adaptations regulating energy and nutrient flow into milk production. Mol Cell Endocrinol. (2023) 559:112078. 10.1016/j.mce.2022.111797 36243202

[23] Fang Y-W Chen S-F Wang M-L Wang M-H. Effects of traditional Chinese medicine-assisted intervention on improving postpartum lactation: a systematic review and meta-analysis. Heliyon. (2024) 10(6):e27154. 10.1016/j.heliyon.2024.e27154 38524574 PMC10957381

[24] Yıldız Birden D Başbuğ A Yurtcu E Kale İ. Implementation of enhanced recovery in women undergoing cesarean delivery improves breastfeeding and maternal perioperative outcomes. Z Geburtshilfe Neonatol. (2025) 229(2):124–30. 10.1055/a-2529-5424 39952278

[25] Onji M Sigl V Lendl T Novatchkova M Ullate-Agote A Andersson-Rolf A. RANK Drives structured intestinal epithelial expansion during pregnancy. Nature. (2025) 637(8044):156–66. 10.1038/s41586-024-08284-1 39633049 PMC11666467

